# Neurocognitive Consequences in Children with Sleep Disordered Breathing: Who Is at Risk?

**DOI:** 10.3390/children9091278

**Published:** 2022-08-25

**Authors:** Pablo E. Brockmann, David Gozal

**Affiliations:** 1 Department of Pediatric Pulmonology, Division of Pediatrics, School of Medicine, Pontificia Universidad Católica de Chile, Santiago 8330074, Chile; 2 Pediatric Sleep Center, School of Medicine, Pontificia Universidad Católica de Chile, Lira 85, 5to piso, Santiago 8330074, Chile; 3 Department of Child Health, Child Health Research Institute, The University of Missouri School of Medicine, Columbia, MO 65212, USA; 4 Department of Medical Pharmacology and Physiology, The University of Missouri School of Medicine, Columbia, MO 65212, USA

**Keywords:** sleep, obstructive sleep apnea, central nervous system, behavior, inattention, neurocognition, biomarkers, phenotypes, precision medicine

## Abstract

Sleep-disordered breathing (SDB) is a prevalent disease in children characterized by snoring and narrowing of the upper airway leading to gas exchange abnormalities during sleep as well as sleep fragmentation. SDB has been consistently associated with problematic behaviors and adverse neurocognitive consequences in children but causality and determinants of susceptibility remain incompletely defined. Since the 1990s several studies have enlightened these associations and consistently reported poorer academic performance, lower scores on neurocognitive tests, and behavioral abnormalities in children suffering from SDB. However, not all children with SDB develop such consequences, and severity of SDB based on standard diagnostic indices has often failed to discriminate among those children with or without neurocognitive risk. Accordingly, a search for discovery of markers and clinically useful tools that can detect those children at risk for developing cognitive and behavioral deficits has been ongoing. Here, we review the advances in this field and the search for possible detection approaches and unique phenotypes of children with SDB who are at greater risk of developing neurocognitive consequences.

## 1. Introduction

Obstructive sleep apnea (OSA) is part of a group of sleep disorders with a broad spectrum of clinical presentations. OSA is the most severe form of this spectrum, which has been coined as sleep-disordered breathing (SDB), and encompasses the occasional presence of snoring, to habitual snoring, upper airway resistance syndrome, obstructive alveolar hypoventilation and of course OSA of different degrees of severity (usually partitioned into mild, moderate and severe) [1]. SDB is among the most prevalent conditions among children, reaching a prevalence up to 18–20% in pediatric populations [2]. Habitual snoring is the main characteristic of this syndrome, which has been associated in numerous studies with a large number of morbid consequences, such as metabolic, cardiovascular, neurocognitive, behavioral, and developmental consequences. In recent years, there has been an increasing interest and drive in establishing methodologies enabling the prediction of neurocognitive consequences associated with SDB [3,4,5,6]. At this point, it should be emphasized that habitual snoring should not be viewed as a normal feature of sleep, and instead should always be seen as a pathological feature of children’s sleep since it may lead to long-term neurocognitive and behavioral consequences [7,8,9]. 

**Figure 1:**
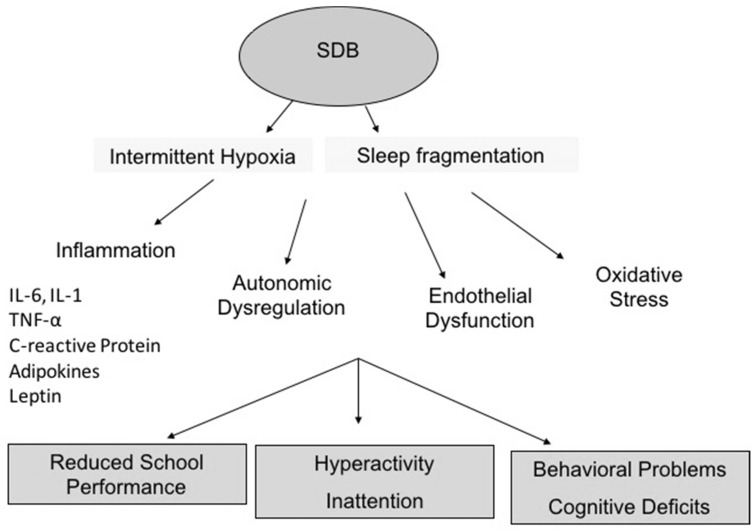
Summary of possible mechanisms involved in SDB-related neurocognitive consequences.

Among the underlying mechanisms which might explain the association between SDB and neurocognitive problems in children, intermittent hypoxia is thought to play an important and critical role in the relationship between SDB and neurocognitive deficits in children [10]. Hyperactive behavior, inattention and reductions in declarative memory capacities, however, have been also detected in children who only had primary snoring, which is unlikely to result in intermittent hypoxia [10,11,12]. On the other hand, respiratory parameters, such as the commonly used apnea and hypopnea indices as well as oxygen desaturation indices have not always emerged as useful or reliable in the prediction of morbidity-related consequences in children with SDB [8,13]. In other words, at any level of SDB severity there will be children who manifest morbid consequences and those who do not. SDB severity is based on the apnea-hypopnea index (AHI) expressed as the number of respiratory events per hour of sleep: primary snoring <1 e/h, mild 1–5 e/h, moderate 5–10 e/h, and severe OSA >10 e/h. However, as the severity of SDB increases the risk probability of altered neurocognitive functioning increases as well [13] for certain cognitive measures while this might not be the case for some behavioral measures [9]. Figure 1 summarizes graphically the possible mechanisms involved in SDB-related neurocognitive consequences. 

Sleep fragmentation resulting from repeated arousals or brief awakenings has been postulated as the other possible mechanism that could be responsible for the neurobehavioral effects associated with SDB. However, changes in sleep architecture detected during standard sleep studies like polysomnography (PSG) have failed to recognize at-risk children [13]. Some efforts to unravel the potential homeostatic alterations in arousals aimed at preserving sleep continuity in the context of the discontinuity introduced by the gas exchange alterations and respiratory efforts triggering activation of arousal centers has provided initial evidence that the global arousal index is not a very useful or valid approach to quantify sleep disruption in the context of pediatric SDB [14,15,16]. Therefore, there was a pressing need to explore more deeply the information embedded in specific patterns of sleep microstructure, such as sleep spindle activity or apply sophisticated deep learning algorithms and artificial intelligence to uncover uniquely predictive biomarkers of cognitive susceptibility in children with SDB [17]. Nonetheless, the search for clinical factors [18,19] that may predict neurocognitive consequences have yielded interesting results to date. Also, the exploration of laboratory biomarkers obtained from serum, urine and breath of affected children may provide correlates of neurocognitive vulnerability [20,21,22]. 

SDB in children is distinct from the OSA that occurs in adults, and such differences are particularly important if we consider that children are in an active phase of myelinization, neural circuitry pruning and expansion, and corresponding neurocognitive development and maturation. Similar to adults, SDB has more than one identifiable clinical phenotype [23,24,25]. In contrast to adults, in whom OSA is predominantly manifest in males, gender distribution may be quite balanced between boys and girls in the prepubertal pediatric population [26]. Considering these differences, SDB-related consequences in children will vary from those seen in adults. Hence, the objective of this review is to analyze current knowledge concerning the association between SDB and neurocognitive consequences in the pediatric age, and to review which new tools for identification of at-risk children may be helpful to refine the identification of at-risk subgroups of SDB children. 

## 2. Characteristics of Neurocognitive Consequences

### 2.1. SDB and School Performance 

SDB has been associated with different forms of neurocognitive consequences in children. Despite the fact that SDB per se was already described more than 130 years ago, and its consequences and associated manifestations were detected a decade later [27], it was not until very recently that SDB was considered to be a relevant medical condition in children [28]. The fist descriptions by Osler already recognized that children with SDB were often “stupid looking” and “slow” [27]. However, it was only in 1976 that the polysomnographically recorded version of OSA was reported in children [28]. It then took two decades until SDB re-emerged not only as a frequent chronic disease in children, but also as a major driver of potentially reversible academic failing performance in primary schools [29]. Indeed, Gozal described that children in the lowest 10th percentile rank of their class, had much higher prevalence of OSA compared with children with higher school grades, and that treatment of OSA in affected children resulted in significant improvements in their school grades [29]. After that study, several other studies from all over the world confirmed this finding [3,4,18,30,31,32]. In a comprehensive systematic review conducted by Galland et al., SDB was found to be significantly associated with lower academic performance in specific areas, especially language, arts, mathematics and science [33]. In that systematic review, lower school grades in specific school subjects were found to be more frequently associated with SDB, than the general average school grades. However, in some of the studies a clear adverse impact of SDB was detected in general school grades [30]. The biological plausibility of such observations was also confirmed by a series of pioneering studies in both adult and developing rodents [34,35,36,37]. In addition, the strength of this association seems to be reinforced by the concurring findings consistently and internationally confirmed across several age groups and cultures. However, variable definitions of both school performance and SDB measures may lead to conflicting results and heterogeneity among the studies. Some studies have used questionnaire-based definitions of SDB, while others have employed polysomnographic definitions based on several different cutoffs. Moreover, neurocognitive consequences have been assessed using several different office-based tests and also by parent or teacher-based reports on school grades. Therefore, a bias may have been introduced in some of these findings. In fact, one of the main problems seems to be the definition of SDB used for the studies that aimed to assess its consequences. 

Besides “real-life” school performance consequences, much more subtle “laboratory” evaluations have strengthened the association between SDB and neurocognitive consequences, as well as other daytime symptoms like excessive sleepiness and behavioral problems. Of note, laboratory evaluations refer to neurocognitive tests conducted by trained psychologist, which aim to investigate in general intelligence quotients and behavioral aspects. These studies will be discussed in the following section.

### 2.2. Intelligence

In 2006, Beebe published a comprehensive review on the association between SDB and neurocognitive and behavioral problems in children [38]. One of the aspects reviewed was the association of the intelligence quotient (IQ) and the presence of SDB. The IQ has been used by most of the studies aiming to assess an association between SDB and neurocognitive consequences. The intellectual ability of children is therefore often measured by several “office-based” based tests like WASI (Wechsler Abbreviated Scales of Intelligence), DAS (Differential Abilities Scale), VMI (Beery Test of Visual-Motor Integration), K-ABC (Kaufman Assessment Battery for Children), CMS (Children’s Memory Scale), or the CPT (Continuous Performance Test). Differences across these tests are often very subtle between children with SDB and controls since after all the comparisons of performance in these tests revolve around children that do not exhibit obvious cognitive deficits or reduced IQ. However, the tests employed by virtually all of the studies are predicated on the major goal of detection obvious developmental delay, i.e., reduced functioning beyond 2 standard deviations below the mean of the normal population. Notwithstanding, Beebe suggested that children with SDB had an overall lower IQ than controls [38]. In summary, results from office-based cognitive tests in children suffering from SDB suggest that most may have some reduction in their cognitive performance that may not necessarily deviate from the normative standards and as such becomes difficult to identify with certainty; in other words, is the IQ of 100 of a child with SDB the result of SDB (in which case the original IQ would be higher) or simply the unchanged and unaffected IQ irrespective of SDB? As mentioned before, the differences in neurocognitive performance obtained in these tests are subtle, and most of the children with SDB function are within the normative range of overall intellectual ability. This fact leads to the question of how should be children at risk of cognitive deficits be recognized if office-based tests can provide evidence of deterioration but only when large cohorts are tested [13] but are unable to detect the affected child in “real-life”?

One of the factors that has been associated with a poorer neurocognitive performance in adults as well as in children is strongly related to poor sleep quality in SDB and manifests as excessive daytime sleepiness. Reduction of sleep duration or disruption of sleep due to SDB has been associated with excessive sleepiness [39,40,41]. In contrast to the initial hypothesis, that children do not exhibit daytime sleepiness like adults with SDB [42], and rather show hyperactive behavior during the day, some recent studies have been able to recognize and document the presence of objective daytime sleepiness in children with SDB, particularly in obese children and children with specific polymorphisms in the gene encoding for tumor necrosis factor α [43,44,45,46,47]. One of the main problems of the assessment of excessive daytime sleepiness in pediatric populations seems to be the fact that the information about its potential presence is often derived from questionnaires filled by the children’s caretakers. Hence, the findings from such questionnaires may be underestimated. In one large community-based study on more than 1000 school-aged children, children with higher scores in a sleepiness questionnaire had significantly lower average mathematics and language grades [32]. In that study, children with SDB had even worse school grades and daytime somnolence [32]. In fact, one very early study in the 1990s by Guilleminault et al. had already associated the presence of SDB with daytime sleepiness [48]. Furthermore, SDB has been postulated a specific cause for excessive daytime sleepiness, as some subtle specific electroencephalographic changes were already identified in patients with SDB and daytime sleepiness [48]. 

### 2.3. Behavioral Consequences 

In the same year 1998 when the association between SDB and poor school performance was highlighted by Gozal [29], Owens et al. showed that parents of children with obstructive sleep apnea reported significantly more frequent daytime externalizing behavior problems in their children, as well as more associated daytime sleepiness [49]. Later on, several studies have focused on the association between SDB and hyperactive/inattentive behavior [50,51,52,53,54].

**Figure 2:**
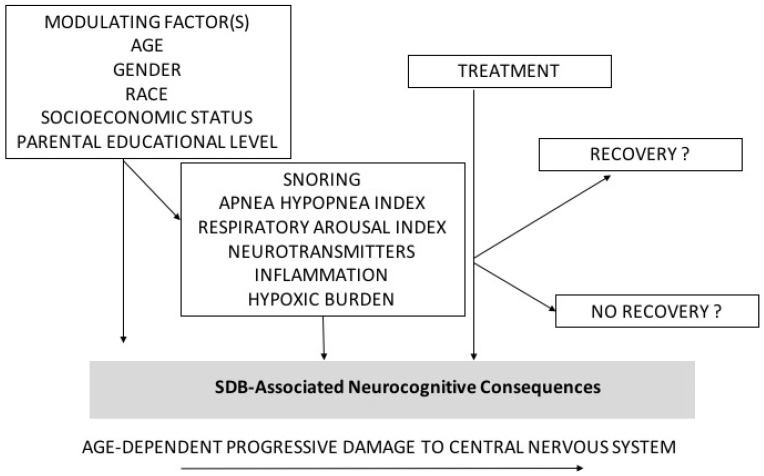
Modulating factors and outcomes after treatment in SDB-related neurocognitive consequences.

The long-term consequences of SDB on children’s behavior were assessed by one study showing that even if SDB was treated or there was an ongoing SDB, several neurocognitive and behavioral domains remained unchanged [55]. Furthermore, in that study, behavioral functioning remained significantly worse in children with SDB than in controls [55]. These findings were predicted by Beebe in his review which indicated the possibility of three post-treatment trajectories in the context of behavioral and neurocognitive consequences of SDB: full recovery, delayed recovery, or residual deficit [38]. Since the evidence points to the fact that SDB causes a period of neurocognitive consequences in a nervous system which is in a critical period of myelinization and development [56,57,58,59,60,61,62,63] after treatment and complete respiratory and sleep architecture recovery from the condition, the above-mentioned scenarios seem to be possible: (i) a “catch-up” and full recovery of the neurocognitive skills of interest; (ii) a loss of function during this developmental period but a resumption of functional catch-up after treatment, leading to a delay; or (iii) a developmental injury that is significantly and permanently irreversible, leading to long-term skill deficits [38]. Reasons for these different outcomes are still unknown. Probably, genetic predisposition, age of treatment, lifestyle factors such as physical activity and diet, and severity of the condition may be crucial factors that influence the final recovery. Hence, prompt treatment may be always necessary, however it still is unknown why SDB may affect more some children than others. Figure 2 summarizes modulating factors and outcomes after treatment in SDB-related neurocognitive consequences. 

### 2.4. Follow-Up Studies and Role of Treatment

Treatment of OSA in children is primarily based on adenotonsillectomy, and in the milder forms anti-inflammatory therapy, such as intranasal corticosteroids or montelukast. The role of treatment has been analyzed in some follow-up studies. In one of the largest, Gozal and Pope analyzed retrospectively data obtained from parents of 13 to 14 y old children [64]. These children were grouped into high-performing and low-performing students (i.e., top and bottom quartiles of academic performance in corresponding classrooms). Students in the low-performing group reported significantly more frequent presence of snoring at ages 2 to 6 years when compared to the high-performing students, and the rates of adenotonsillectomy for snoring were also higher in the low-performing group [64]. 

In a large multi-center study and the only randomized controlled study of OSA to date, the CHAT study, 464 children were followed up to 7 months after adenotonsillectomy conducted for treating their OSA [65]. In that study, the impact of early adenotonsillectomy was compared to a strategy termed as “watchful waiting”. Although polysomnographic indices normalized in 79% of the children undergoing adenotonsillectomy, no significant improvements in office-based neuropsychological test results were found [65]. In the CHAT study, only reduction in symptoms, and some improvements in secondary outcomes concerning behavior, and quality of life emerged in the early adenotonsillectomy group [65]. Despite some limitations of that study (e.g., the severity of OSA was restricted and children with persistent oxygen desaturation were excluded for ethical reasons, and the study did not include preschool children in whom SDB is especially important); the CHAT study highlights some critical aspects concerning the limitations of polysomnographic indices for diagnosis and follow-up of SDB. The search for markers that may identify which patient with SDB is at-risk for developing neurocognitive consequences will be discussed later in this review.

In a 20-year follow-up study using telephonic surveys of adults who had been diagnosed with OSA during childhood, those with a diagnosis of severe OSA as children had significantly lower academic degrees than their peers [66]. Thus, long-term sequelae of SDB during childhood seem to be present and relevant, even after treatment. In summary, there is consistent evidence that supports the relationship between SDB and neurocognitive consequences in children. This evidence is now based on meta-analyses, systematic reviews, clinical and laboratory-based studies and has been replicated in a wide range of ages, ethnicities, and different countries and cultural and socioeconomic backgrounds. Since the evidence supporting such relationship seems quite clear, the mechanisms that are involved should be actively sought after. 

## 3. Mechanisms Mediating Neurocognitive Consequences in SDB

### 3.1. Intermittent Hypoxia

As SDB primordial physiological disturbance consists in the occurrence of intermittent increases in upper airway resistance that may progress to complete upper airway obstruction, increased vibratory elements develop (snoring) during high resistance states and a secondary reduction in available oxygen supply along with increased accumulation of carbon dioxide manifest systemically. As such, it is not surprising that an increasing interest on intermittent hypoxia has emerged as one of the major determinants of SDB-related consequences. A systematic review published some years ago regarding the association between SDB and intermittent hypoxia, of the 55 articles that met inclusion criteria, 43 (78.2%) reported the presence of an adverse effect concerning intermittent hypoxia and SDB [67]. That review compared the effect of chronic intermittent hypoxia in congenital heart disease, asthma, respiratory diseases and SDB, and found several studies that showed deleterious consequences in development, behavior, and academic achievement in children [67]. As mentioned above, a large body of bench-based and animal model experiments have further investigated the potential roles played by intermittent hypoxia mimicking OSA and the mechanistic pathways mediating such effects. Overall, it has become apparent that among many other likely pathways, those involving inflammation and oxidative stress are clearly critical for the CNS consequences imposed by OSA [68,69,70,71,72].

### 3.2. Arousal and Sleep Fragmentation

As children suffering from SDB without intermittent hypoxia may also manifest significant neurocognitive consequences [4,50,73] the search for other perturbations highlighted the role of what has been called “sleep fragmentation” [74]. Sleep fragmentation can occur as classical cortical arousals, subcortical arousals, or as autonomic activation (also called autonomic arousals). The first type of arousal are characterized by disruption of the sleep electroencephalography (EEG) and of the normal sleep architecture, which can be detected by EEG and has clear standard definitions [75]. Cortical arousals have been invoked as mediating neurocognitive consequences in children, probably due to recruitment of systematic inflammatory processes [76,77,78]. As current guidelines and criteria for scoring arousals may not be recognizing more subtle “subcortical events”, some studies have tried to detect these events in milder forms of SDB, such as in primary snoring. These sub-cortical alterations may trigger an autonomic activation that leads to changes in heart rate, blood pressure, and other manifestations [79,80,81]. This autonomic activation—in addition to cortical arousal—is possibly associated with neurocognitive consequences in apparently milder forms of SDB [74]. 

Sadeh et al. reported that frequent sleep disruption and fragmentation in children [82] was associated with emotional information processing during early adolescence. Classical sleep fragmentation as documented by the prototypic changes in the sleep EEG is however not always present in children with SDB. Therefore, the search for more subtle markers for arousal has been developed in recent studies. Among the studied markers of sleep disruption, the cyclic alternating pattern of EEG has been postulated as a possible mechanism for sleep disruption in children with SDB [83]. In one study that specifically assessed differences in the cyclic alternating pattern in children with SDB compared with healthy controls, [83] children with mild SDB showed a decreased cyclic alternating pattern rate [83]. Kheirandish-Gozal et al. postulated that a lower cyclic alternating pattern (i.e., altered sleep microarchitecture) was a marker of subtle sleep disruption that is not recognizable by classic arousal scoring definitions, and that this disruption may lead to neurocognitive and behavioral dysfunction in children with SDB.

In a more recent study by Gutierrez-Tobal et al., overnight EEG spectral activity and nonlinear metrics of irregularity were strongly correlated with neurocognitive consequences of SDB in children [84]. It is therefore possible that multidimensional parameterization of mathematically derived markers from the sleeping EEG will ultimately enable development of a quantitative composite marker of cognitive risk obtained from the sleep EEG during standard diagnostic polysomnography.

Another specific marker that is susceptible to sleep disruption seems to be the density and patterning of sleep spindles. Sleep spindles are bursts of EEG activity with a frequency range between 11 and 16 Hz (usually 12–14 Hz) with a duration of 0.5 s or greater (usually 0.5–1.5 s) [85]. Several studies have correlated the presence and frequency of sleep spindles to various neurocognitive domains, such as memory consolidation [86,87,88,89], development of social skills [90], intelligence [91], and overall intellectual capability [88,89,91,92,93,94]. Brockmann et al. have demonstrated that sleep spindles are associated with working memory [95] in children. In that study, sleep spindles were significantly different between children with mild SDB compared with healthy peers. Moreover, sleep spindles were associated with IQ, and several other neurocognitive domains in that cohort of children [95]. As sleep microstructure seems to have emerged as a possible tool for recognizing sleep disruption in children with SDB, we launched later on a study aiming to assess the possible differences in sleep spindle activity between children with the mildest form of SDB (i.e., primary snoring) and controls [96]. In that study, we found that children with primary snoring exhibit reduced spindle activity compared with their healthy peers. Even in the absence of apneic and hypoxemic events, sleep microarchitecture as measured by sleep spindle activity was significantly different throughout NREM sleep stages in children with SDB [96].

Altogether, there seems to be striking evidence for the role of sleep microstructure and specifically sleep spindles as markers of sleep disruption. Future studies should address the question about the association of these more subtle forms of sleep disruption with memory-related neurobehavioral processes, involving, among others, the thalamocortical neural circuitry [97]. 

### 3.3. Molecular Basis of Neurocognitive Damage

The next step to gain insights into possible mechanisms that link SDB and neurocognitive consequences in children was to assess a molecular basis responsible for end-organ damage, in this case, targeting the central nervous system. Therefore, many studies have analyzed in recent years the role of inflammation, endothelial dysfunction, and biomarkers as possible links between SDB and neurocognitive consequences. 

In 2019 a systematic review analyzed current knowledge concerning the potential biomarkers of SDB-related neurocognitive impairments in children [98]. In that review, plasma insulin growth factor-1 (IGF-1) and Alzheimer’s disease (AD)-related biomarkers were found to have a significant role as markers of neurocognitive damage in children with SDB. Most biomarkers reported were blood or plasmatic molecules [98]. De Luca Canto reviewed also potential SDB related biomarkers and OSA-associated morbidities; plasma IL-6 and high sensitivity C-reactive protein were among the most interesting molecules found to be associated with adverse consequences [99]. In that review, and specifically concerning neurocognitive consequences, however, alterations in a set of urinary neurotransmitters emerged as the most plausible biomarker panel for identifying children at-risk [99]. Urinary biomarkers are particularly attractive in children since they may be easily obtained and are not painful. Kheirandish-Gozal et al. went even a step further in the search for possible biomarkers for neurocognitive damage [21]. They aimed to find neurotransmitters excreted in urine, with the hypothesis that these neurotransmitters have a major signaling role in the nervous system and that they may be potentially affected by SDB in children [21]. In that study, urinary catecholamines, taurine and GABA predicted neurocognitive consequences in children with SDB [21]. A combinatorial diagnostic approach using specific cutoffs for the above-mentioned neurotransmitters was proposed as a tool for identifying children with OSA and the risk for developing neurocognitive consequences. A similar approach predicated on differential expression of urine proteins also yielded very promising results [100]. 

In summary, several putative biomarkers have been evaluated in children with SDB. Future use of specific cutoffs of the urinary or plasmatic levels of these biomarkers as composite panels may permit the screening of children with SDB and identify those at-risk for neurocognitive consequences. 

## 4. Discussion and Future Directions

Finally, after reviewing the current evidence that clearly links SDB with neurocognitive consequences in children and the possible mechanisms that may lead to this damage, the question concerning who is at risk arises. Is there a test, clinical feature, laboratory marker that predicts in whom diagnosis and treatment is urgent? Are there possible identifiable phenotypes that are helpful in identifying a worse evolution of SDB?

Not all children with SDB develop neurocognitive consequences, hence the search for a possible diagnostic tool for at-risk patients is urgently needed. In line with this, one of the logical approaches was to assume that a more severe form of SDB would lead to more severe forms of neurocognitive consequences. There is, however, conflicting evidence on this hypothesis. In a large, community-based study on school-aged children Hunter et al. demonstrated that children with a higher apnea hypopnea index showed significantly lower cognitive performance [13]. Children with more severe disease based on this polysomnographic measure had consistently lower scores in several neurocognitive tests. However, the statistically significant differences in office-based cognitive tests were subtle, as many children in all severity groups scored within the normal range. On the other hand, stratified analyses also identified that attention was specifically affected in habitually snoring children with normal polysomnographic indices (i.e., primary snorers, the mildest form of SDB) [13]. Hence, the reasons for neurocognitive damage in this mild group evaded the potential utility of polysomnographic indices as a predictor of morbidity [13]. Nonetheless, the fact that a cut-off revolving around the apnea-hypopnea criterion separating mild from moderate OSA was also the cut-off in which the probability of cognitive deficits increased substantially provides an important justification to the implementation of surgical therapy for children in the moderate to severe OSA range, since the risk-benefit of surgery reaches equipoise to the possible sequelae of OSA [13].

The problem concerning the detection of children at-risk for neurocognitive consequences, however, persists. Considering that the most prevalent forms of SDB are primary snoring and milder forms of OSA, predictive usefulness of polysomnographic indices seem to fail in these groups. In a study using a prediction model, based on a simple parental questionnaire, some items showed acceptable accuracy for identification of snoring children with poor school performance [19]. Some oximetric and polysomnographic indices emerged also as predictors of poor school performance, however, each index alone showed only weak correlations, and only after a statistically constructed model, diagnostic accuracy was enhanced [19]. 

Going even further in the search for markers of neurocognitive risk among children with SDB, Smith et al. demonstrated that snoring was a predictor of behavioral outcomes, even after adjusting for polysomnographic indices such as the apnea hypopnea index [8]. There was also a dose-effect model observed in the degree of snoring and behavioral outcomes in that study [8]. This study may, hence, provide an explanation for the considerable neurocognitive consequences found in children with primary snoring and milder forms of SDB. Addition of urinary biomarkers and some of the aforementioned plasmatic molecules may be useful in the identification of neurocognitive consequences in children with SDB [99]. Future studies should address the question of a combinatory approaches to determine which clinical, questionnaire-based, biomarkers, and some polysomnographic features are useful and clinically practical tools to detect at-risk children with SDB.

## 5. Conclusions

SDB has been associated with serious and long-term neurocognitive consequences in children. Today, there is striking cumulative evidence of the existence of this association and a multitude of studies conducted worldwide support this finding. Since the early studies in the 1990s, much has been discovered concerning the mechanisms and pathways that link SDB with several neurocognitive domains. However, which clinical, laboratory or polysomnographic measures are clinically useful for identification of those children with SDB at-risk for developing neurocognitive consequences remains very poorly defined. Thus, efforts to identify the vulnerable phenotypes of SDB and confirm that timely treatment may diminish or prevent SDB associated long-lasting damage to the central nervous system in children are amply justified. This is undoubtedly a needed task for future studies, as this research may help to improve the health and well-being of a large number of children.
